# Cardiosphere-derived cells in the primary prevention of sepsis-induced acute lung injury in pigs

**DOI:** 10.1371/journal.pone.0338336

**Published:** 2026-01-27

**Authors:** James Frederick Dawkins, Asma Nawaz, Lizbeth Sanchez, Ralf Sulague, Aiyana Verfaillie, Juan Cardenas, Eugenio Cingolani, Michael Lewis, Eduardo Marbán

**Affiliations:** 1 Smidt Heart Institute, Cedars-Sinai Medical Center (CSMC), Los Angeles, California, UNITED STATES OF AMERICA; 2 Pulmonary/Critical Care Div., Dept of Medicine (CSMC), Los Angeles, California, UNITED STATES OF AMERICA; Versiti Blood Research Institute, UNITED STATES OF AMERICA

## Abstract

Acute respiratory distress syndrome (ARDS) prevention in patients coincide with significant risk factors, including severe sepsis. Cardiosphere-derived cells (CDCs) are cardiac stromal/progenitor cells with anti-inflammatory and immunomodulatory effects, which may impact favorably on sepsis-induced acute lung injury (ALI). We used a pig model of sepsis (lipopolysaccharide, LPS) to test whether CDCs, given IV in a preventive paradigm, can ameliorate ALI, relative to placebo. Yorkshire/Landrace hybrid domestic pigs (n = 34) were divided into 3 groups: 1) healthy controls; 2) LPS (60ug/kg) + saline (placebo) group; and 3) LPS + CDC group, with CDCs given in 3 different doses (25, 50 and 100 million cells) after LPS. All pigs were briefly intubated on mechanical ventilation on room air to obtain baseline and 1-hour data and again at the final endpoint of 48hours. Chest mechanics, gas exchange, hemodynamics, blood, bronchoalveolar lavage assays and lung histology were assessed. LPS + CDCs compared with LPS + saline improved: gas exchange (increased hypoxemia ratio [p = 0.03],chest mechanics (respiratory compliance [p = 0.002]; peak and plateau pressures [p = 0.01/0.049]), pulmonary hemodynamics (decreased pulmonary artery pressures [p = .0008]), reduced BAL neutrophils [p = 0.04]), and systemic cytokines, e.g., IL-1, IL-6, IL-18 and IFNγ [p < 0.05]) and serum creatinine (p = 0.02), while alveolar lavage cytokines, e.g., IL-1, IL-6,IL-8,and IL-18 were changed. Histopathology was improved (decreased atelectasis, hemorrhage and arteriolar thickness). In summary, CDCs given in a preventive fashion, attenuated manifestations of LPS-induced ALI. As CDCs have an extensive safety record in hundreds of patients, our findings motivate clinical testing of CDCs for the primary prevention of sepsis-induced ARDS.

## Introduction

Clinical risks for acute respiratory distress syndrome (ARDS) include severe sepsis, large volume blood transfusions, and polytrauma [1]. A classic study evaluated 695 ICU patients and found that 26% developed ARDS, in which sepsis was the most common risk factor (43% of the ARDS cohort) [1]. In other studies, 25–62% of patients with severe sepsis developed acute lung injury (ALI) [2–4]. Mortality in these studies was high (35.5–40%). Despite advances in the supportive care of ARDS (lung protective ventilation, prone positioning, enhancing ventilator synchrony, fluid restrictive strategies etc. [5]), mortality remains high (~40%) [5–7]. Pharmacologic and other treatments in established ARDS have failed to significantly impact the primary disease process [6,7]. A large number of infectious and non-infectious conditions may be complicated by ARDS [5,8]. Amongst these, sepsis and non-pulmonary sources remain important risk factors.

Given the refractory nature of established ARDS, the pendulum has moved towards the concept of prevention in patients with significant risk factors [6,8,9]. Primary prevention emphasizes the early recognition of risk factors such as severe sepsis, while secondary prevention starts during early ALI to offset the development of full-blown ARDS [6,8,10]. Pharmacologic approaches to primary prevention failed [11,12]. In secondary prevention, the use of high flow nasal cannula oxygen and a small phase 2a study of inhaled therapies (corticosteroid + beta agonist) had some positive results [13,14]. The use of cell therapy is potentially attractive, as it may tackle multiple aspects of the underlying pathobiology [15]. Cardiosphere-derived cells (CDCs) are heart stromal/progenitor cells which are in advanced clinical development (phase 3) for Duchenne muscular dystrophy [16,17]. Via the release of exosomes which contain highly bioactive cargoes (especially non-coding RNAs), CDCs act in a paracrine fashion to exert potent anti-inflammatory, immunomodulatory, anti-fibrotic, and anti-apoptotic effects [18,19] which may be beneficial in sepsis. We thus hypothesize that CDCs, given early in sepsis, will blunt the pathobiologic drivers that lead to ALI/ARDS, ameliorating outcomes. In a pig model of sepsis (induced by lipopolysaccharide; LPS), we tested the ability of CDCs, given in a preventive paradigm, to improve gas exchange, chest mechanics and lung inflammation.

## Methods

### Full methodological details are provided in the online supplement

All studies were performed within the guidelines of the US animal welfare act The experimental protocol (IACUC 9300−53 total animals) was approved by the Institutional Animal Care and Use Committee at Cedars-Sinai Medical Center. LPS injury is a validated model for ALI/ARDS in pigs [20,21]. Animals were monitored daily, and euthanized while under general anesthesia at the endpoint diagnostic procedure with a veterinary euthanasia solution, or KCL given to effect. Humane endpoints or conditions for humane euthanasia were identified if an animal was in systemic shock, while demonstrating neurologic signs or severe dyspnea. All animals in this study were euthanized at the 48-hour endpoint.

### Study design

**Fig 1:**
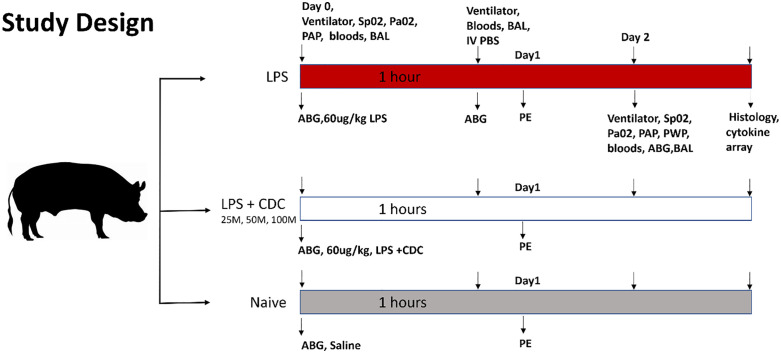
Study design. Study design of an acute lung injury model in pigs. Pig were randomly assigned into 5 groups and received LPS (n=11), LPS + CDCs at 3 different doses (25M n=6, 50M n=6, 100M n=5 cells total), or naïve pigs (n=4) were given PBS alone. Diagnostics were repeated at different intervals, baseline, 1 hour after LPS or saline infusion, and at the 48 hour endpoint. Diangostics included arterial blood gases, measures of respiratory phyisology (PA pressure, P/F ratio, compliance, plateau pressure, peak pressure, PCO2) peripheral venous blood sampling, bronchoalveolar lavage. Following euthanasia, endpoint lung tissue was harvested for histologic analysis.

Thirty-four domestic pigs (35–45 kg) were enrolled in 3 cohorts: healthy “naïve” pigs with no injury (naïve, n = 4), a control group that received LPS (60ug/kg Lipopolysaccharides from *Escherichia coli* O55:B5, Sigma) in 250 ml saline (LPS, n = 11), and an active treatment group that received LPS, followed by one of 3 doses of human CDCs (25M- n = 6, 50M n = 6; 100M n = 5), given intravenously (IV). Arterial blood gases (ABGs), and biomarkers from venous blood and bronchoalveolar lavage fluid (BAL) were obtained (**Fig 1**). Animals were briefly intubated to obtain baseline measurements and then extubated and recovered on room air, but re-intubated to acquire the final 48-hour data collection.

### Pig procedures

On day 0, pigs were anesthetized using propofol infusion and intubated. Mechanical ventilation using a clinical grade ventilator with 21% O_2_ was employed. Exhaled tidal volume (Vt), minute ventilation, respiratory frequency, peak end expiratory pressure, peak pressure, plateau pressure, driving pressure, and respiratory system compliance were recorded continuously **(Fig 1).** Next, standard bronchoalveolar lavage (BAL) was performed on 2 occasions using phosphate buffered saline (PBS, 10 ml) instilled into the right lower lobe with aspiration after ~1 minute. Vascular sheaths were implanted in the femoral artery and vein. A total of ~30mL of venous blood, and <2ml total of arterial blood, was drawn during experimental course. A diagnostic catheter was then advanced into the pulmonary artery (PA) for pressure measurements. LPS was then infused IV over 1 hour. After LPS delivery, animals received IV infusion of PBS (20 ml) or CDCs in PBS (20 ml), over 15 minutes. An additional group of “naive” animals received neither LPS nor CDCs. At 48 hours, access and diagnostic procedures were repeated, followed by euthanasia and necropsy **(Fig 1).**

### Respiratory physiology

Animals were ventilated on room air at sea level. Vt was set at 10 ml/kg of body weight (veterinary short-term standard for healthy pigs). Peak and plateau pressures, peak pressure, and respiratory compliance were measured at each timepoint. PaO2/FiO2 ratio (P/F) as well as PCO_2_ were calculated from ABGs. Pulmonary arterial pressure was measured by pulmonary artery catheterization.

### Lung pathology

Lung tissue samples were taken from each lung lobe. Tissue from the lower lobes of each pig were used for comparison. Tissue was cut and fixed for hematoxylin and eosin (H&E) staining.

### Renal pathology

Bilateral kidney samples were taken from the glomerular area of the kidneys. Tissue was cut and fixed for H&E and picrosirius red staining.

### Cardiosphere-derived cell culture

Human CDCs were isolated and prepared from heart biopsies as previously described [22].

### Immunology of CDCs in an LPS pig model

#### Hematology/Biochemistry.

Complete blood counts, including differential cell counts, were performed on ADVIA hematology analyzers (ANTECH diagnostics®).

#### BAL.

Nucleated cell counts and erythrocyte counts in lavage fluid were determined by a hemacytometer.

#### Multiplex analysis of cytokines.

Luminex xMAP technology was used for quantification of 13 porcine cytokines, chemokines, and growth factors. Serum and BAL samples were acquired at baseline (T1), and at endpoint prior to sacrifice (T3).

## Results

### Physiologic effects of CDCs

**Fig 2:**
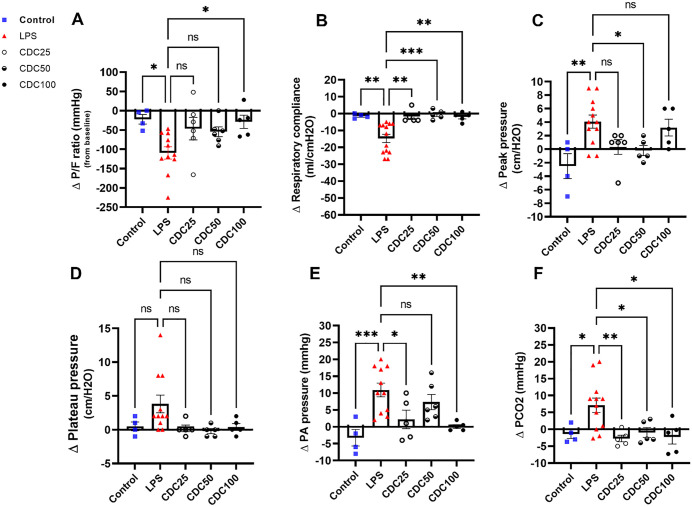
A- CDC injection attenuated a decrease in P/F ratio following LPS injury, which was most evident at the 100M dose. (LPS-CDC^25^ -46.67±71.39, LPS-CDC^50^ -54.67±31.16 LPS-CDC^100^ -28.8±38.43 vs. LPS -109.4±52.97, p=0.03). **B-** Following suit, acute changes in compliance were preserved in CDC and saline injected pigs, but not in LPS only animals. (LPS-CDC^25^ -1.8±3.89ml/cmH2O, LPS-CDC^50^ -0.600±2.70ml/cmH2O, LPS-CDC^100^ -2.00±5.64ml/cmH2O vs. LPS -14.77±8.79ml/cmH20, p=0.002. **C-** We observed differences in peak pressure in 2 of the 3 CDC injected groups (LPS-CDC^25^ 0.33±2.65cm/H2O, LPS-CDC^50^ -0.20±1.64cm/H2O, LPS-CDC^100^ 3.20±2.77cm/H2O vs LPS 4.08±3.37cm/H2O, p=0.01) **D-** while changes in plateau pressure were preferable in the CDC injected groups (LPS-CDC^25^ 0.20± 1.09cm/H2O, LPS-CDC^50^ -2.0±0.83cm/H2O, LPS-CDC^100^ 0.400±1.14cm/H2O, LPS 3.81±4.35cm/H20, p=0.049) **E-** Pulmonary pressure was held closer to baseline or reduced in most groups except for LPS only (LPS-CDC^25^ 2.2±6.14mmhg, LPS-CDC^50,^ 7.33±±5.50mmhg, LPS-CDC^100^ 0.10±1.24mmhg, LPS 10.91±6.71mmhg, p=0.0008). **F-** Gas exchange was more efficient in all groups as changes in CO2 were improved compared to LPS (LPS-CDC^25^ -2.7±2.1mmhg, LPS-CDC^50^ -0.92±3.25mmhg LPS-CDC^100^ -2.24±4.74mmhg vs. LPS 7.11±7.42mmhg, p=0.003. (D values represent baseline D0, and endpoint, D2).

Respiratory function was preserved in all 3 animal dosing groups receiving CDCs compared to LPS only. Data is represented as the change (Δ) between baseline values, D0, and endpoint, D2 (**Fig 2**). The reduced P/F ratio in LPS only pigs was attenuated in animals receiving CDCs, at any dose (p = 0.03). No intergroup dose differences were noted in pigs receiving CDCs; however, the high dose (LPS-CDC^100^) group trended most favorably. The protective effect was such that LPS + CDC pigs were statistically equivalent to naïve healthy pigs, p = 0.03 (**Fig 2A**). Similarly, respiratory compliance was preserved in LPS + CDC pigs (p = 0.002; **Fig 2B**). Peak pressure was unchanged in two of the three CDC dose subgroups, but not in LPS + placebo (p = 0.01; **Fig 2C**). Plateau pressure was preserved in all LPS + CDC dose groups compared to LPS alone. (p = 0.049; **Fig 2D**)**.** Prevention of elevated pulmonary artery systolic following LPS was evident in the LPS-CDC^25^ and LPS-CDC^100^ groups (p = 0.0008; **Fig 2E**). There was no difference in right atrial pressures between the groups. On day 1, LPS animals exhibited labored breathing compared to CDC treated groups. For PCO2, LPS + CDC pigs were like healthy control animals, in contrast with LPS only animals (p = 0.003; **Fig 2F**), (**S2 Table**). In summary, LPS + CDCs compared with LPS + saline improved gas exchange, chest mechanics and pulmonary hemodynamics.

### Histopathology

**Fig 3:**
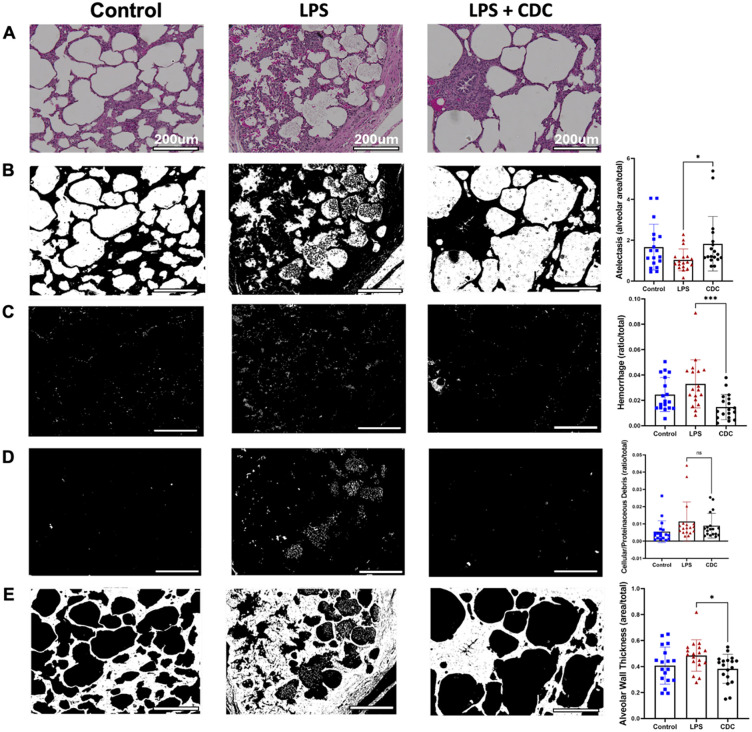
Automated MATLAB analysis of H&E stained lung sections. **A-** Representative H&E sections of randomly selected slides from the porcine lung. **B-** Mask of control (left) LPS (middle) and LPS+CDC (right). Pigs suffering from LPS induced lung injury had the severity of atelectic injury greatly attenuated by administration of CDCs. Healthy animals with no lung injury demonstrated an alveolar area of 1.665±1.120area/total, LPS injured animals demonstrated collapsed alveoli 1.043±0.543area/total, while CDC treated pigs demonstrated the most preserved alveolar space 1.825±1.330area/total.(p=0.02) **C-** Mask analysis of hemorrhagic damage at endpoint. LPS injury alone caused significant bleeding within the lung parenchyma 0.0329±0.019area/total where this was rescued in LPS injured pigs treated with CDC’s, 0.014±0.001area/total (p=0.001).**D-** Next, cellular and proteinaceous debris was evaluated. Although there was a non statistical difference between groups, LPS pigs treated with CDC’s demonstrated a healthier trend when compared to LPS injury alone, CDC 0.008±0.007area/total vs. LPS 0.01134±0.01138, p=ns . **E-** Lastly, Alveolar wall thickness in CDC pigs more closely reflected healthy alveoli of naïve pigs, Naïve 0.4070±0.1427area/total, LPS 0.4847±0.1213area/total, CDC 0.3825±0.1116area/total (p=0.01). Magnification 10x, scale bar 200um.

Quantitative lung histopathology analysis performed on H&E-stained slides (naïve n = 3, LPS n = 3, LPS + CDC n = 3). Histology showed much improved atelectasis in LPS + CDC pigs compared to LPS (p = 0.02; **Fig 3B**). Additionally, hemorrhage within the lung parenchyma was severe in LPS + placebo pigs, with strong attenuation in LPS + CDC pigs (p = 0.001; **Fig 3C**). There was a trend for reduced cellular debris in the CDC group, but the differences were not significant compared to LPS + placebo (p = ns; **Fig 3D**). Lastly, marked alveolar wall thickening, observed in LPS only pigs, was prevented by CDCs (p = 0.01; **Fig 3E**). A gross representation of *en bloc* lung tissue after LPS or LPS + CDC is shown in **S1 Fig**. Acute histopathologic lung injury score demonstrated prevention of further parenchymal lung damage in LPS + CDC treated pigs, p < 0.0001 (**S2 Fig****).** In summary, LPS + CDCs compared with LPS + saline improved several histopathological features of ALI.

### The effects of CDCs on systemic and alveolar immune cells

**Fig 4:**
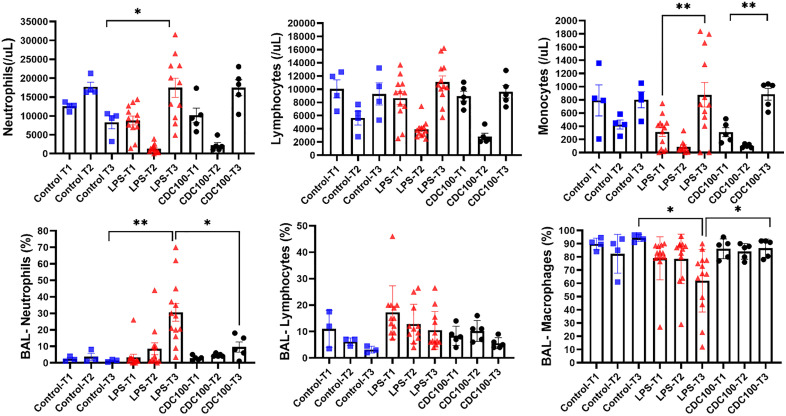
Peripheral blood mononuclear cell concentration over the course of the study at 3 timepoints (T1-T3). **A-** Neutrophil concentration at baseline was similar in all groups. In LPS pigs, there was a shift out of peripheral circulation immediately following LPS administration, however this rebounded by the 48 hour endpoint (T3). **B-**Lavage samples demonstrated an overall increase in neutrophil population in LPS pigs from T1-T3. This was attenuated at endpoint in the LPS-CDC^100^ group compared to LPS pigs (LPS-CDC^100^ 9.5±6.87%,LPS 30.58±10.43%, p=0.03). **C-D** Lymphocyte activity was similar between all groups in both blood and lavage samples, p=ns. **E-** A peripheral monocytosis can be observed in all LPS injured groups over the course of the study, however there were no differences at endpoint. **F-** Alveolar lavage samples showed a progressive decrease in macrophage population in LPS injured pigs, however this was rescued in the LPS-CDC^100^ group`(LPS-CDC^100^ 88.7±6.87%, LPS 62.00±23.61%, p=0.037).

Regarding immune cell behavior, the CDC^100^ group demonstrated the largest physiologic response **(Fig 2).** We thus decided to focus our assays on this treatment group. In healthy control animals, systemic neutrophil levels increased by 30% between baseline (T1), and immediately following sham infusion of saline (T2; p = 0.007). At 48 hours, neutrophil levels had returned to baseline values (p = ns; T1 vs T3). By contrast, pigs injected with LPS + placebo showed a decrease in neutrophils immediately following LPS infusion (p < 0.0001), followed by a rebound neutrophilia at endpoint to levels higher than baseline (p = 0.004). A similar pattern was observed in CDC injected pigs, with no difference at endpoint between groups that received LPS **(Fig 4A)**.

A different pattern was apparent however in BAL fluid. While the neutrophil population in healthy controls remained consistent over 48 hours, LPS pigs displayed an immediate increase in neutrophils which likely reflected chemotaxis-induced neutrophil migration. LPS pigs showed an increase in concentration from baseline to endpoint. This migration was curbed in the LPS-CDC^100^ group where at endpoint alveolar neutrophils were decreased relative to LPS alone (p = 0.038). The magnitude of increase from baseline to endpoint was also greater in LPS pigs (p < 0.0001) vs. LPS-CDC^100^, p = ns **(Fig 4B)**. Peripheral lymphocytosis was observed in the LPS group between baseline and endpoint, [p = ns], with no appreciable changes in the CDC or PBS groups in peripheral blood or lavage fluid **(Fig 4C–4D)**. This contrasts with a few consistent changes in alveolar lymphocytes (p = 0.07; **Fig 4E**). Monocyte recruitment was increased in groups that received LPS (LPS + placebo, p = 0.007; LPS + CDC^100^, p = 0.0009) while the healthy control group remained unchanged. There was a relative decrease in macrophages in LPS pigs (p = 0.04), except for the LPS-CDC^100^ group (p = ns; **Fig 4F**). In summary, LPS + CDCs compared with LPS + saline showed somewhat discordant results between serum and BAL assays. Key and important findings were a decrease in BAL neutrophils and increased BAL macrophages in the CDC treated animals.

### Cytokine expression in alveolar lavage fluid

**Fig 5:**
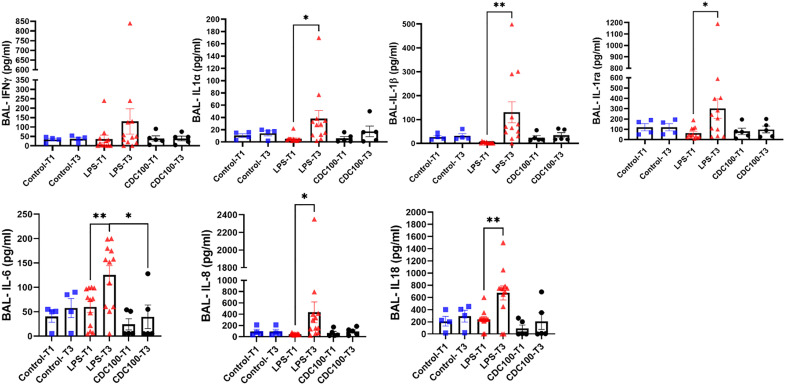
Cytokine expression in alveolar lavage fluid. Multiplex array for cytokine quantification lung lavage samples. **A-** IFNγ was increased in BAL samples of LPS only pigs (LPS, baseline, 36.25±67.62pg/ml, endpoint 129.6±234.3pg/ml, p=ns) . There was no difference over the course of the experiment in healthy animals (baseline, 32.50±14.5pg/ml, endpoint, 37.00±17.45pg/ml, p=ns), or LPS+CDC pigs (baseline, 39±31.44pg/ml, endpoint, 38.8±27.03pg/ml, p=ns) **B-** Inflammatory cytokine IL-1α significantly increased in BAL samples from LPS only pigs (baseline, 5.27±5.39pg/ml, endpoint 37.93±46.20pg/ml, p=0.02). Small changes in IL-1α were observed in CDC injected pigs (baseline, 6.00±17.28pg/ml, endpoint 17.28±19.63pg/ml p=ns. **C-** IL-1β was found elevated in LPS only injured pigs (baseline, 2.89±3.43pg/ml, endpoint 130.3±152.0pg/ml, p=0.0083). **D-** In our LPS injured pigs, there was an increase in IL-1ra (baseline, 63.13±55.15pg/ml, endpoint, 301.3±330.6pg/ml, p=0.021. Lavage samples showed an attenuated IL-1ra response in healthy pigs and LPS+CDC group. **E-** Inflammatory cytokine IL-6 was increased in LPS lavage fluid (baseline, 59.82±.39.07pg/ml, endpoint, 125.6±66.02, p=0.0071). Injection of CDCs to LPS injured pigs reduced IL-6 concentration in alveolar samples . **F-** In alveolar lavage fluid, LPS injured pigs showed a large increase in IL-8 concentration (baseline,46.63± 12.34pg/ml, endpoint 432.9±640.8pg/ml, p=0.048), while CDC treated pigs demonstrated the opposite **G-** 48 hours after LPS injury IL-18 increases substantially in alveolar fluid (baseline, 239.3±152.4pg/ml, endpoint, 676.7±406.5pg/ml, p=0.0021). This occurrence is present in a small percentage of CDC pigs (baseline, 93±126.9pg/ml, endpoint 211±.306.6pg/ml, p=ns).

In addition to immune cell activation and proliferation following LPS injury, a coinciding shift in cytokine expression was evident in LPS injured pigs. BAL samples were evaluated for intragroup changes from baseline, and endpoint in healthy, and in LPS injured groups + /- CDC intervention. The increased neutrophil infiltration in the lungs could potentially cause the release of certain cytotoxic inflammatory proteins contributing to additional tissue damage. In lavage fluid we observed an increase in IFNγ in LPS only animals (p = ns), however this was attenuated in CDC treated groups (p = ns) **(Fig 5A)**. Pro-inflammatory cytokines IL1α (p = 0.02) **(Fig 5B)**, and IL1β (p = 0.0083) **(Fig 5C)** were also increased in LPS only pigs, but this was not observed in CDC treated animals. This finding parallels a prevention of macrophage depletion in tissue of CDC treated pigs. Anti-inflammatory cytokine IL1ra was increased in both LPS injured groups (LPS, p = 0.021, LPS + CDC, p = ns) **(Fig 5D)** which suggest the normal inhibitory mechanism of IL1ra binding nonproductively to the receptor utilized by the IL-1 family. As a result of decreased pulmonary macrophage population in the LPS only group, IL-6 was increased significantly following LPS injury (p = 0.0071), but this effect was not observed in animals receiving CDCs (p = ns) **(Fig 5E)**. Additional causation of the lack of IL-6 mobilization in CDC treated pigs is the attenuated immune response and cellular protection conferred following protective intervention. Increased expression of IL-8 is supportive of the severe neutrophil infiltration observed in LPS only pigs (p = 0.048), but again, this effect was not observed in LPS injured pigs treated with CDCs (p = ns) **(Fig 5F)**. As a member of the IL-1 family of cytokines, IL-18, a prominent cytokine associated with acute lung inflammation, is steadfast following LPS injury (p = 0.0021) and diminished in the face of LPS injury followed by CDCs (p = ns) **(Fig 5G)**. In summary, LPS + CDCs compared with LPS + saline BAL assays showed distinct trends for reduction in most cytokines assayed (with values virtually identical to those in healthy control pigs) and a significant decline in IL-6.

### Cytokine expression in serum

**Fig 6:**
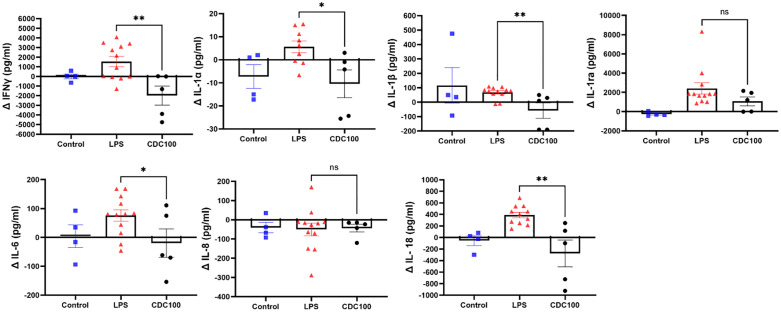
Cytokine expression in serum. **A-** IFNγ was increased in serum of LPS pigs (LPS, Δ1540±1829pg/ml, vs LPS+ CDC, -Δ1987±2219pg/ml p=0.003). **B-** Inflammatory cytokine IL-1α was significantly increased in LPS serum samples (LPS, Δ5.61±7.65pg/ml, vs LPS+CDC -Δ10.41±13.5pg/ml, p=0.014). **C-** Following suit, IL-1β increased in LPS only serum samples, (LPS, Δ67.5±39.92pg/ml, vs LPS+CDC -Δ58±121.3pg/ml, p=0.0048). **D-** In our LPS injury model we observed an increase in IL-1ra LPS pigs in serum samples. In serum LPS, (LPS, Δ2401±2049pg/ml, vs LPS+CDC ±1063±1031pg/ml, p=0.19). **E-** Inflammatory cytokine IL-6 was increased in serum in LPS pigs and was reduced in LPS+CDC pigs (LPS, Δ75.93±69.48pg/ml, vs LPS +CDC -Δ19.87±109.9pg/ml, p=0.04). **F-** Both groups demonstrated a decrease in IL-8 from baseline to endpoint in serum (LPS, -Δ50.17±113pg/ml, vs LPS+CDC 43.3±44.32, p=0.8). **G-** 48 hours after LPS injury IL-18 increases substantially in systemic circulation. This phenomenon is opposite in CDC pigs as there is either no change, or a decrease in IL-18 from baseline to endpoint (LPS, Δ389.5±163.1pg/ml, vs LPS+CDC -Δ277±520pg/ml p=0.0014).

The focus of this study is to evaluate the effects of CDCs on acute sepsis, and the manifestation of acute lung injury. However, given the disease model in which LPS was given systemically, we evaluated changes in serum cytokine expression in parallel with alveolar fluid. An intergroup comparison between healthy, and LPS + /- CDCs was evaluated. IFNγ is an important T-cell mediated cytokine that can contribute to the pathogenesis of acute lung injury. When observed from baseline to the terminal endpoint, IFNγ expression was reduced in the CDC group, while a notable increase was observed in the LPS only group (p = 0.003) **(Fig 6A)**. The IL-1 family of cytokines (IL-1α, p = 0.014, IL-1β, p = 0.0048, IL-1ra, p = ns) followed a similar trend, with the exception of anti-inflammatory cytokine IL-1ra, in which LPS injured groups demonstrated increased expression from baseline to endpoint **(Fig 6B–6D)**. Anti-inflammatory cytokine IL-6 was increased systemically in the LPS only injury group, however not in LPS + CDC group, a sequala to the attenuated inflammatory response of CDC treated animals (p = 0.04) **(Fig 6E)**. Both injury groups showed a decrease in IL8 from baseline to endpoint (p = ns) (Fig 6F), however, 48 hours after LPS injury, IL-18 increases substantially in animals injured by LPS without CDCs (p = 0.0014) **(Fig 6G).** In summary, LPS + CDCs compared with LPS + saline serum assays showed significant decrements in several cytokines (INFγ, IL- α, IL-1β, IL-6 and IL-18) with a trend to reduction in IL-12ra.

### CDCs prevent renal azotemia following LPS injury

**Fig 7:**
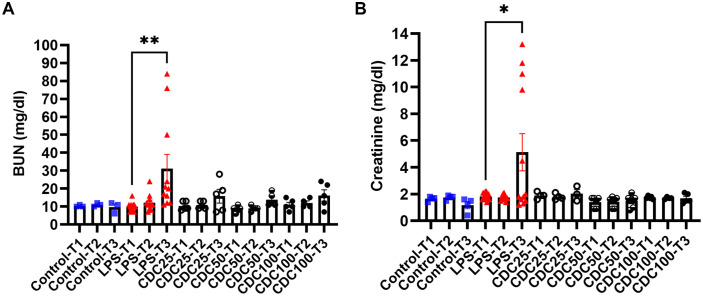
Clinical evaluation of renal azotemia. **A-**Peripheral blood samples were obtained and serum isolated. Across the study, there was no increase in BUN exept for in the LPS only group (T1 9.92±2.36mg/dl, vs, T3 31.18±26.47mg/dl, p=0.008. **B-**serum creatinine followed suit with a significant increase in the LPS only group between baseline and the 48hr endpoint (T1 1.83±0.24mg/dl, vs, T3 5.12±4.80mg/dl, p=0.02).

Acute kidney injury is associated with sepsis through multiple mechanisms including the impact of [23,24].pro-inflammatory cytokines in renal cells [24–26]. As a surrogate to kidney injury, we evaluated parameters of renal dysfunction including blood urea-nitrogen (BUN), and creatinine. Normal healthy controls and CDC treated animals at various doses maintained normal renal function during the 48-hour period, unlike pigs that were injected with LPS + saline, where renal dysfunction was evident by increments in BUN, p = 0.008 **(Fig 7A)**, and creatinine, p = 0.02 **(Fig 7B)**, suggesting a systemic impact of CDCs on the kidney. Renal histopathology was performed. A total renal injury score demonstrated that renal injury was reduced in LPS-CDC pigs compared to LPS injury alone (**S2**–S3 **Fig**). In summary, LPS + CDCs compared with LPS + saline in serum assays and histology showed improvement in renal function and less acute kidney injury.

## Discussion

Even in the modern era of supportive treatment advances in ARDS, the condition still has an unacceptably high mortality rate [5–7,27]. Sepsis is an important risk factor for severe ARDS [1]^,^ Thus, early and effective treatment to offset ALI/ARDS (apart from antibiotics and source control) is attractive. In a preventive strategy, the window to act is narrow: in one trial, the median time from trial entry to full-blown ARDS was only 20 hours [25]. A treatment modality that addresses many of the aberrations underlying sepsis-induced ALI/ARDS would thus be an attractive and major adjunctive advance. Here we examined the ability of CDCs, given in a preventive paradigm, to attenuate the development of overt ARDS. Early therapy with CDCs prevented key physiological, cellular, and biochemical abnormalities that characterize ALI/ARDS; indeed, many of these measures remained at or near control levels. CDC administration improved gas exchange, chest mechanics, and pulmonary hemodynamics, while reducing pulmonary inflammation and preserving renal function. These findings have important clinical implications for primary prevention of sepsis induced ALI/ARDS.

Sepsis caused by bacteria and other organisms induces a cascade of events resulting in widespread injury and sequelae including ALI/ARDS, with the host and microbial genomes undergoing a “genome war” involving immune reprogramming [15]. In brief, bacteria and other microbes are sensed by toll -like receptors which are expressed on lung and immune cells, as part of the initial innate immune response. Stress responsive transcription factors then activate pro-inflammatory cytokines, chemokines and other inflammatory factors [15]. Damage to lung endothelial and epithelial cells promotes loss of barrier integrity, while uncontrolled inflammation contributes to, oxidative stress, complement activation and microvascular thrombi. For primary or secondary preventive measures to be effective, therapies geared to offset the development of ALI/ARDS need to act simultaneously on multiple different pathways to preserve barrier integrity.

LPS (also called endotoxin) is a component of the outer membrane of gram-negative bacteria. Such bacteria account for two thirds of bacterial sepsis [1]. With systemic administration, lung endothelial cells are the initial target [26,30]^.^ We chose the LPS model of ALI/ARDS model as it simulates an important cause, produces a picture similar to ARDS, has similar pathophysiologic sequelae to clinical ALI/ARDS and is reproducible [20]. We chose a large animal model over a rodent one, which commonly have reduced sensitivity to endotoxemia. Pigs and sheep are more sensitive to LPS than dogs [27]; the pig model in general tends to more closely approximate human ARDS [28–30].

CDCs are cardiac stromal cells that secrete exosomes [31–33] containing cargos of bioactive nucleic acids, notably including ncRNA such as microRNAs and Y RNAs [19,32,33]. Exosome cargo, and therapeutic potency, vary according to the cell type of origin, with CDC-EVs being more effective in cardioprotection compared with mesenchymal stem cells [34]. CDCs have potent anti-inflammatory properties in several preclinical disease models [17,35–38], inhibiting many proinflammatory cytokines [18,32,39]. The anti-inflammatory effects of CDCs can be largely explained by their effects on macrophages which can be polarized to anti-inflammatory phenotypes, and which play an important role in early ARDS pathogenesis [16,33–35,40]. Further, CDCs augment activity of the Nrf2 antioxidative pathway to reduce oxidative stress [35], and they are potently anti-fibrotic [22,36,41–43], which may offset the fibroproliferation seen in ARDS.

### Study limitations

Our work has several notable limitations: 1) The major objective of this study was to test the hypothesis that CDCs have disease-modifying bioactivity in a large-animal model of ARDS, based on the known anti-inflammatory properties of the cells. While we performed consistency checks regarding the underlying mechanism, our work was not designed to provide new insights into the mechanism of action of CDCs. 2) This study did not include continuous mechanical ventilation over 48 hours. Rather, the very short-term intubations allowed easy measurements of pulmonary hemodynamics and acquisition of BAL procedures only. We thus simulated a non-intubated state of sepsis, to discern CDC treatment effects at a preventive stage of progression to ARDS. 3) The use of tidal volumes of 10 ml/kg was chosen based on short-term veterinary anesthesia protocols to prevent atelectasis. We believe the very short durations of mechanical ventilation likely averted meaningful volutrauma and injury. 4) As we chose a model of essentially spontaneously breathing pigs for both groups, the issue of developing ventilator-associated pneumonia was largely mute and prophylactic antibiotics were therefore judged unnecessary. 5) The elimination half-lives of pro-inflammatory cytokines are often rather short. For example, serum levels of IL-6 peaked at 12 hours in an inflammatory pre-clinical model [44]. Thus, we may have missed significant and important changes in cytokines that evolved between 1 and 48 hours. Further, discordances between serum and BAL cytokine levels have been reported in ARDS and severe pneumonia [45–47]. Contributors may have included our technique; BAL volumes use and sampling error. However, it should be emphasized that trends in the same direction were observed for our BAL assays compared to our serum data.6) Additionally, our study did not address early secondary prevention. 7) The dose finding component of this study was limited to small groups of animals (n = 5–6), this was due to feasibility and the pilot nature of the dose finding component of the study. We believe that our selected dose to investigate in detail (100M CDC’s) demonstrated the most physiologic and biochemical benefits in this disease model following treatment.

## Conclusion

We have demonstrated in a pig LPS model the power of CDCs administered intravenously to markedly attenuate many aspects of ALI in a primary preventative paradigm. These salutary impacts give motivation to test whether CDCs might be useful to offset ALI/ARDS in patients with severe sepsis, given that CDCs have a strong track record of safety in clinical trials.

## Supporting information

S1 Fig Gross swine lung appearance at 48hour endpoint following A- Saline infusion, B- LPS infusion, C- LPS infusion follwed by 100M CDC delivery. (DOCX)

S2 Fig Acute lung injury score (LPS, 11.28 ± 1.841, CDC, 8.61 ± 1.501, p < 0.0001). Acute kidney injury score (LPS, 9.5 ± 2.85, CDC, 6.3 ± 2.0, p = 0.0059).
(DOCX)

S3 Fig Automated MATLAB histopathologic analysis of the porcine kidney. **A-B** Representative H&E and picrosirius red stained slides from the bilateral sections of the glomerular area of the porcine kidney. **C-** Mask of control (left) LPS (middle) and LPS + CDC (right). Pigs suffering from LPS injury had a slight increase in Bowman’s space and renal tubular area, but the severity was not much different between LPS injured groups (LPS 0.1783 ± 0.06, CDC 0.1531 ± 0.11, p = ns). **D-** Mask analysis of hemorrhagic damage at endpoint. LPS injury alone caused significant bleeding within the kidney glomerulus and prevented in LPS injured pigs treated with CDCs, (LPS, 0.0036 ± 0.002, CDC, 0.0007 ± 0.0009, p = 0.0009). **E-** Cellular and proteinaceous debris deposition was then evaluated. Here, there was a non-statistical difference between groups, however LPS pigs treated with CDCs demonstrated a healthier trend with less cell debris, when compared to LPS injury alone, LPS 0.003 ± .0018, CDC 0.0018 ± 0.0017, p = ns. **E-** Lastly, collagen deposition in LPS injured pigs trended more poorly than CDC. Magnification for H&E is 10x, scale bar 200um, and picrosirius red is 4X. scale bar 1000um.
(DOCX)

S1 Table H&E staining, manufacturers, catalog numbers and protocol. (DOCX)
