# Supplementary material for: Cardiosphere-derived cells in the primary prevention of sepsis-induced acute lung injury in pigs
Source: PLoS One. 2026 Jan 27;21(1):e0338336. doi: 10.1371/journal.pone.0338336 (PMC12843593; doi:10.1371/journal.pone.0338336)
Supplement: S1 Fig — (DOCX) [file pone.0338336.s001.docx]

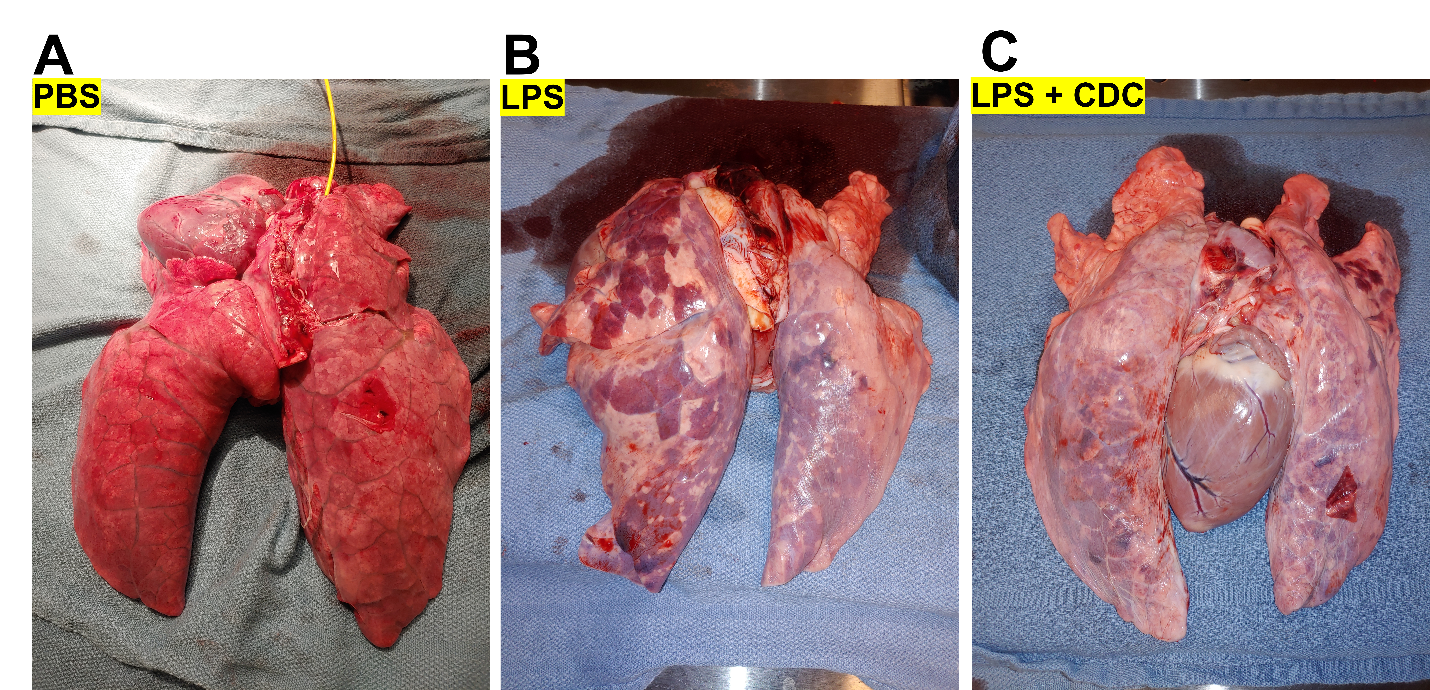
 **Figure S1:** Gross swine lung appearance at 48hour endpoint following **A-** Saline infusion, **B-** LPS infusion, **C-** LPS infusion follwed by 100M CDC delivery.
