# Supplementary figures and images for: Cardiosphere-derived cells in the primary prevention of sepsis-induced acute lung injury in pigs
Source: PLoS One. 2026 Jan 27;21(1):e0338336. doi: 10.1371/journal.pone.0338336 (PMC12843593; doi:10.1371/journal.pone.0338336)

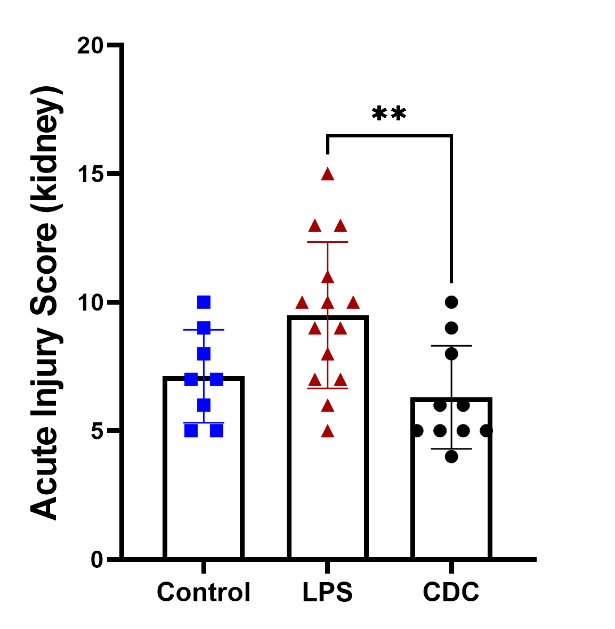

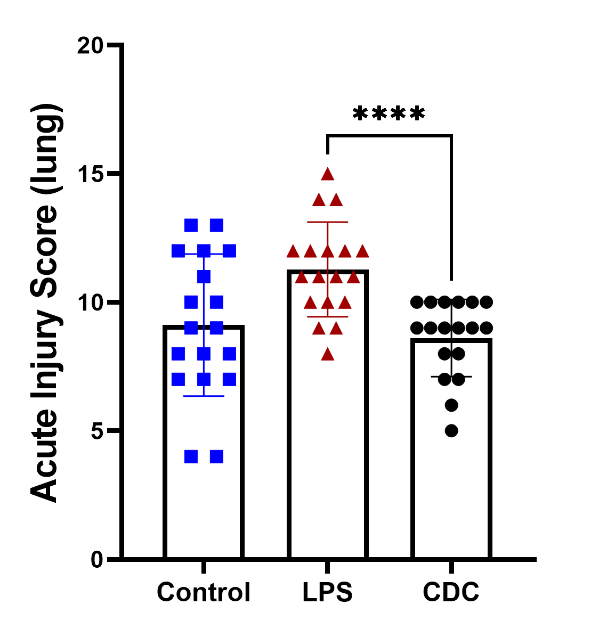


**Figure S2:** Acute lung injury score (LPS, 11.28±1.841, CDC, 8.61±1.501, p<0.0001). Acute kidney injury score (LPS, 9.5±2.85, CDC, 6.3±2.0, p=0.0059).

Supplement: S2 Fig — Acute kidney injury score (LPS, 9.5 ± 2.85, CDC, 6.3 ± 2.0, p = 0.0059). (DOCX) [file pone.0338336.s002.docx]
