# Supplementary material for: Cardiosphere-derived cells in the primary prevention of sepsis-induced acute lung injury in pigs
Source: PLoS One. 2026 Jan 27;21(1):e0338336. doi: 10.1371/journal.pone.0338336 (PMC12843593; doi:10.1371/journal.pone.0338336)
Supplement: S3 Fig — A-B Representative H&E and picrosirius red stained slides from the bilateral sections of the glomerular area of the porcine kidney. C- Mask of control (left) LPS (middle) and LPS + CDC (right). Pigs suffering from LPS injury had a slight increase in Bowman’s space and renal tubular area, but the severity was not much different between LPS injured groups (LPS 0.1783 ± 0.06, CDC 0.1531 ± 0.11, p = ns). D- Mask analysis of hemorrhagic damage at endpoint. LPS injury alone caused significant bleeding within the kidney glomerulus and prevented in LPS injured pigs treated with CDCs, (LPS, 0.0036 ± 0.002, CDC, 0.0007 ± 0.0009, p = 0.0009). E- Cellular and proteinaceous debris deposition was then evaluated. Here, there was a non-statistical difference between groups, however LPS pigs treated with CDCs demonstrated a healthier trend with less cell debris, when compared to LPS injury alone, LPS 0.003 ± .0018, CDC 0.0018 ± 0.0017, p = ns. E- Lastly, collagen deposition in LPS injured pigs trended more poorly than CDC. Magnification for H&E is 10x, scale bar 200um, and picrosirius red is 4X. scale bar 1000um. (DOCX) [file pone.0338336.s003.docx]

**
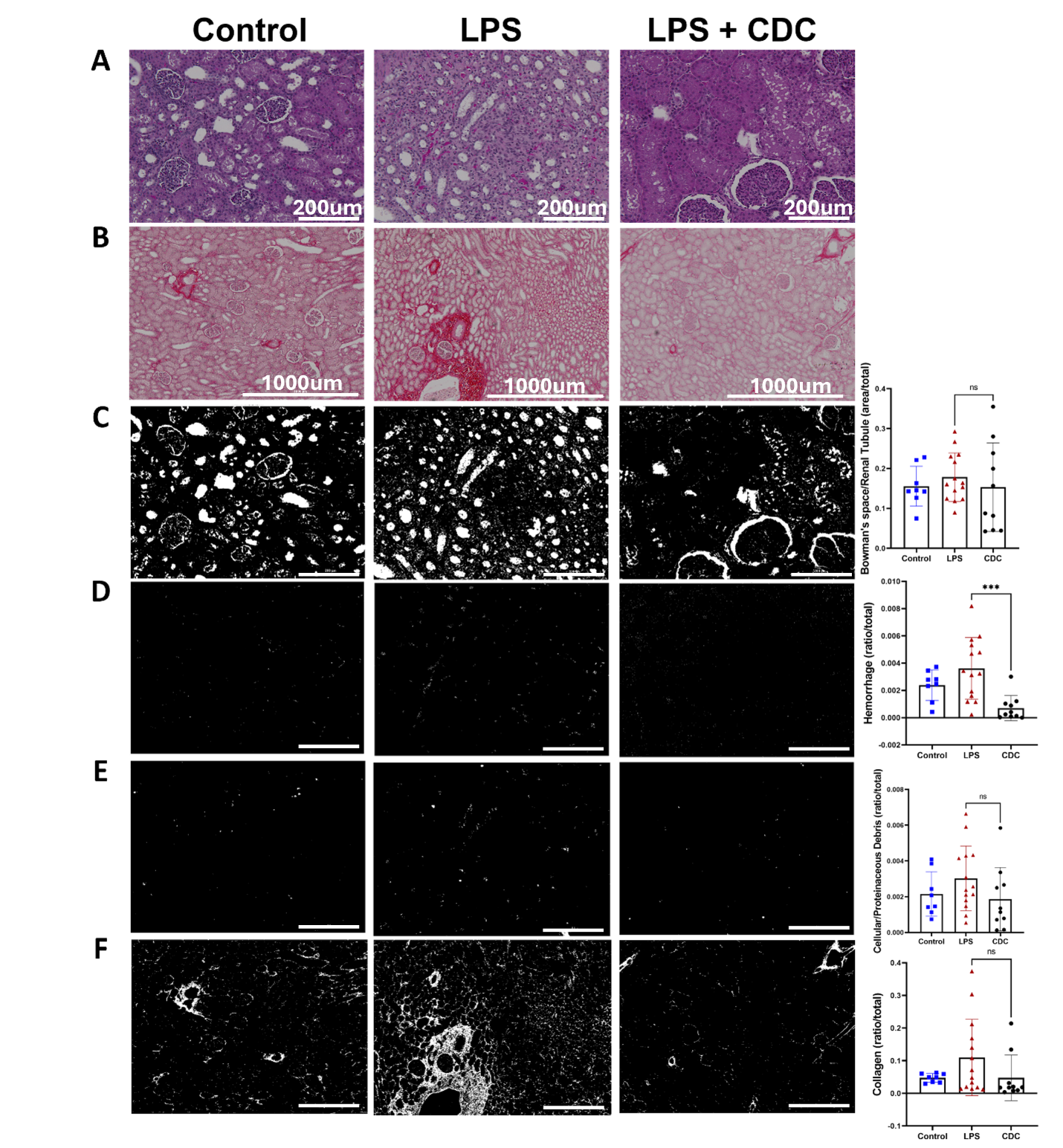
**

**Figure S3:** Automated MATLAB histopathologic analysis of the porcine kidney. **A-B** Representative H&E and picrosirius red stained slides from the bilateral sections of the glomerular area of the porcine kidney. **C-** Mask of control (left) LPS (middle) and LPS+CDC (right). Pigs suffering from LPS injury had a slight increase in Bowman’s space and renal tubular area, but the severity was not much different between LPS injured groups (LPS 0.1783±0.06, CDC 0.1531±0.11, p=ns). **D-** Mask analysis of hemorrhagic damage at endpoint. LPS injury alone caused significant bleeding within the kidney glomerulus and prevented in LPS injured pigs treated with CDCs, (LPS, 0.0036±0.002, CDC, 0.0007±0.0009, p= 0.0009). **E-** Cellular and proteinaceous debris deposition was then evaluated. Here, there was a non-statistical difference between groups, however LPS pigs treated with CDCs demonstrated a healthier trend with less cell debris, when compared to LPS injury alone, LPS 0.003±.0018, CDC 0.0018±0.0017, p=ns. **E-** Lastly, collagen deposition in LPS injured pigs trended more poorly than CDC. Magnification for H&E is 10x, scale bar 200um, and picrosirius red is 4X. scale bar 1000um.
