# Supplementary material for: Cardiosphere-derived cells in the primary prevention of sepsis-induced acute lung injury in pigs
Source: PLoS One. 2026 Jan 27;21(1):e0338336. doi: 10.1371/journal.pone.0338336 (PMC12843593; doi:10.1371/journal.pone.0338336)
Supplement: S1 Table — (DOCX) [file pone.0338336.s004.docx]

**Table S1: H&E staining, manufacturers, catalog numbers and protocol**

| Materials | Catalog number |
| --- | --- |
| IMDM media | Fisher Scientific #  12440079 |
| 2-Mercaptoethanol | Fisher Scientific #21985023 |
| Penicillin-Streptomycin solution 10,000 units | Sigma-Aldrich #p4333 |
| CryoStor cell cryopreservation media | Sigma-Aldrich #C2874 |
| Cell culture bag Permalife PL07 | Origene #PLO7 |
| Heparin |  |
| Nitroglycerin  40um cell strainer | Fisher Scientific #08771 |
| Cryomed freezer | Fisher Scientific #508866-151 |
| Xylene | 2 minutes |
| Xylene | 2 minutes |
| 100% ethanol | 2 minutes |
| 100% ethanol | 2 minutes |
| 95% ethanol | 2 minutes |
| Water wash | 2 minutes |
| Hematoxylin | 3 minutes |
| Water wash | 1 minute |
| Differentiator (mild acid) | 1 minute |
| Water wash | 1 minute |
| Bluing | 1 minute |
| Water wash | 1 minute |
| 95% ethanol | 1 minute |
| Eosin | 45 seconds |
| 95% ethanol | 1 minute |
| 100% ethanol | 1 minute |
| 100% ethanol | 1 minute |
| Xylene | 2 minutes |
| Xylene | 2 minutes |
| Coverslip | DPX |
